# Measuring inequality in community resilience to natural disasters using large-scale mobility data

**DOI:** 10.1038/s41467-021-22160-w

**Published:** 2021-03-25

**Authors:** Boyeong Hong, Bartosz J. Bonczak, Arpit Gupta, Constantine E. Kontokosta

**Affiliations:** 1 Marron Institute of Urban Management, New York University, New York, NY USA; 2 Stern School of Business, New York University, New York, NY USA; 3 Center for Urban Science and Progress, New York University, Brooklyn, NY USA

**Keywords:** Psychology and behaviour, Environmental health, Society

## Abstract

While conceptual definitions provide a foundation for the study of disasters and their impacts, the challenge for researchers and practitioners alike has been to develop objective and rigorous measures of resilience that are generalizable and scalable, taking into account spatiotemporal dynamics in the response and recovery of localized communities. In this paper, we analyze mobility patterns of more than 800,000 anonymized mobile devices in Houston, Texas, representing approximately 35% of the local population, in response to Hurricane Harvey in 2017. Using changes in mobility behavior before, during, and after the disaster, we empirically define community resilience capacity as a function of the magnitude of impact and time-to-recovery. Overall, we find clear socioeconomic and racial disparities in resilience capacity and evacuation patterns. Our work provides new insight into the behavioral response to disasters and provides the basis for data-driven public sector decisions that prioritize the equitable allocation of resources to vulnerable neighborhoods.

## Abstract (web summary)

Understanding how cities respond to extreme weather is critical; as such events are becoming more frequent. Using anonymized mobile phone data for Houston, Texas during Hurricane Harvey in 2017, the authors find that mobility behavior exposes neighborhood disparities in resilience capacity and recovery.

## Introduction

Cities face significant risks from extreme weather events, sea level rise, and heat waves associated with anthropogenic climate change. Between 1980 and 2000, there were a total of 51 major hurricanes, severe storms, and flooding events in the United States, resulting in more than $254 billion in damage and the loss of 1331 lives^1–3^. Since 2000, the number of these events has more than doubled, to 125, with damage exceeding $1 trillion and loss of life increasing approximately five times^1–3^. While the impacts of the growing frequency and devastation of climate hazards are being disproportionately felt in coastal cities^4–13^, urban policy-makers and emergency responders have few tools to understand patterns of evacuation, impact, and recovery at high spatial and temporal resolutions^9,14,15^. Such granular information could be used to evaluate the effectiveness of, and disparities in, local evacuation behavior and to help fully understand the recovery lifecycle of communities, which in turn could support localized need-based resource allocation and long-term planning strategies.

While the concept of resilience has been defined and measured across a range of scientific disciplines, such as biology, material science, psychology, ecology, and engineering^16–22^, few studies examine urban resilience within a framework of complex adaptive systems. Here we focus on an integrated, socio-behavioral definition of community resilience across temporal and spatial scales, namely the ability of a complex urban system—characterized by the nonlinear interactions of social, environmental, and physical subsystems—to withstand and rapidly recover from an extreme event, including natural or man-made disasters^16–24^. While conceptual definitions provide a foundation for the study of disasters and their impacts, the challenge for researchers and practitioners alike has been to develop objective and rigorous measures of resilience that are generalizable and scalable, taking into account spatiotemporal dynamics in the response and recovery of localized communities^21^.

Emerging sources of large-scale mobility data can be used to model human behavior in response to natural disasters. Accurate assessment of spatiotemporal mobility patterns of people in cities could provide new insights into many urban operational and planning decisions, such as traffic forecasting, resource allocation, crisis and outbreak prediction, and disaster management^25–28^. By extension, the application of geo-tagged big data in this context can move us toward more robust, validated urban dynamics models that can begin to account for the socio-ecological complexities unique to the urban environment^10,15,26,29–32^. For example, mobile phone data offer the opportunity to understand detailed human mobility patterns in cities at unprecedented resolution^26–31^. In particular, large-scale geo-tagged information has been especially useful for travel demand estimation and inferring land use patterns at granular spatial resolutions^26,29^.

Despite the rapid growth of big data and urban computing, applications to the fields of urban resilience and disaster management are limited and piecemeal^21,33,34^. Previous empirical studies have been constrained by data limitations and inconsistent and diverging indicators of resilience^24^. In the case of hurricanes, for example, studies have focused on descriptive, citywide evacuation statistics and correlative factors of evacuation by using post-disaster surveys with relatively small samples, such as the National Household Travel Survey^26,29,35–41^. In an effort to capture the spatiotemporal dynamics of event response, some studies have used social media data like Facebook or Twitter to understand disaster-related online behavior changes and detect crisis regions during natural disaster events^15,42–47^. While research using these digital traces can help to measure the overall impact of a disaster, social media data are characterized by representativeness bias and often require aggregate spatial resolutions, such as the county or city scale, to capture sufficient geo-tagged samples^15,42–47^. Despite the increased interest in mobility data by scholars in disaster management fields, limited attention has been paid to neighborhood-level evacuation and recovery patterns at scale and the disparate behavioral responses across communities with divergent socioeconomic and demographic characteristics.

Understanding hyper-local disaster response is an important foundation for effective and equitable community planning and urban resilience strategies. Spatiotemporal evacuation and recovery patterns, represented by mobility dynamics before, during, and after a disaster, are directly connected to the socio-behavioral resilience of urban systems. This requires the identification and quantification of emergent mobility networks within and across neighborhoods impacted, directly or indirectly, by an event of sufficient magnitude to disrupt normal activity patterns. Since individuals and neighborhoods represent interconnected social and physical urban systems, their dynamics at high spatiotemporal resolution can signal local distress, growth, and recovery. This information can be used to assess disaster-related impacts and community resilience at scale and thus inform both operational emergency management decisions and long-range community planning and preparedness.

In this paper, we develop and demonstrate a generalizable method using large-scale smartphone geolocation data to measure and evaluate neighborhood response and recovery to natural disasters. We utilize Hurricane Harvey as a case study. Hurricane Harvey struck Houston, Texas in August 2017, resulting in the second costliest disaster in U.S. history with at least $125 billion in damage due to extreme flooding, described as an unprecedented 1000-year flood event^48,49^. We analyze geolocation data derived from smartphone applications over a 2-month period (August 1, 2017 to September 30, 2017) covering ~1 million unique users in Houston, equivalent to ~35% of the total population of the metropolitan area. We measure and analyze community resilience by estimating variations in resident mobility patterns as a proxy for human behavior and social activity, a central indicator of urban system dynamics, before, during, and after Hurricane Harvey. The findings provide insight into the timely evaluation of disaster impact and time-to-recovery for individual communities, as well as the correlates of neighborhood resilience capacity. We highlight the disparities in evacuation and recovery patterns associated with varying socioeconomic, demographic, and geophysical community attributes.

## Results

After data preprocessing in order to ensure data confidentiality (see Methods and Supplementary Information for a detailed description of the data and data processing workflow), we focus exclusively on data points representing smartphone activity falling within the boundaries of Harris County (central Houston, including the Downtown Houston area) for the 2-month period between August 1st, 2017 and September 30th, 2017. For the purpose of this study, we assume that a single device represents an individual. We begin by identifying daily neighborhood residential activity levels using 829,350 unique devices, representing ~35% of the Houston population. We define neighborhoods by using ~4500 one-kilometer by one-kilometer (0.6214 mile × 0.6214 mile) grid cells and each ping location from an individual device is assigned to the corresponding neighborhood grid cell based on its location.

The spatial distribution of activity before, during and after the hurricane clearly demonstrate the impact of the event on mobility behavior, as shown in Supplementary Fig. 1. During the pre-impact period (Supplementary Fig. 1a), activities are heavily concentrated along major roadways and in the Downtown Houston area. This pattern is disrupted when Hurricane Harvey hits the area (Supplementary Fig. 1b), resulting in significantly lower levels of activity scattered across the city. We also observe that activity patterns gradually return to pre-impact levels after the hurricane (Supplementary Fig. 1c).

### Neighborhood clustering and community resilience capacity

**Fig. 1:**
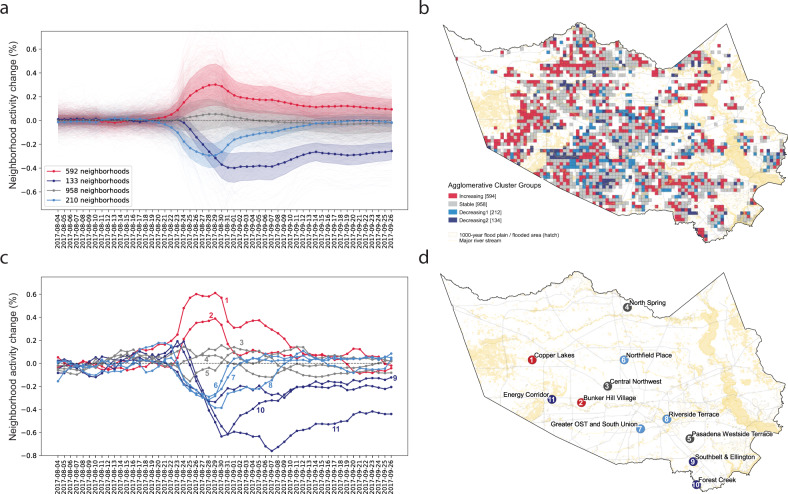
Distinct neighborhood groups based on disaster response and recovery patterns as identified by agglomerative clustering. **a** This time series plot shows community activity level trends over the time period from August 4th through September 26th. The agglomerative clustering algorithm identifies four distinct groups that represent similar patterns of disaster response and recovery at the neighborhood level. Plots denote mean activity of each cluster with one standard deviation uncertainty band. **b** The map shows spatial patterns of identified clusters. Most dark blue neighborhoods show a significant decrease in community activities due to devastating flooding near reservoir areas. **c** Illustrative neighborhood activity curves for each cluster group associated with their location in (**d**).

To understand the disparities in disaster response and recovery patterns across neighborhoods, we classify neighborhoods into four groups based on changes in community activity levels over time using an unsupervised machine learning technique. We implement an agglomerative hierarchical clustering algorithm to identify similarities in neighborhood-level response behaviors. Figure 1 illustrates the clustering results. Notably, we observe empirical activity curves that reflect theoretical resilience and recovery patterns^16,17,20,21,50^. The curves clearly demonstrate four phases of disaster response: pre-event equilibrium, event impact, recovery, and post-event equilibrium, as shown in Fig. 1a and Supplementary Fig. 2. While conceptual resilience curves were developed through disaster scenario simulations or sampled survey data, our findings demonstrate the potential of high spatiotemporal data to quantify hyper-local resilience patterns derived directly from observed mobility trajectories.

We utilize these empirical resilience curves to quantify localized Harvey impacts and community resilience capacity. We also introduce a unified metric of resilience capacity that integrates both the magnitude of impact and recovery duration, calculated as the area under/over the curve (shadowed in Supplementary Fig. 3).

### Neighborhood response and recovery profiles

We are able to determine a range of neighborhood impact and recovery profiles. As illustrated in Fig. 1, activity in Groups 1 and 2 form U-shape curves, representing a decrease in regular community activity levels during the hurricane. Activity levels in these neighborhoods return close to (but distinct from) pre-event levels during the post-Harvey equilibrium period. As shown in Table 1, the average magnitude of impact for Group 1 and Group 2 is 0.48 and 0.34, respectively, meaning these two neighborhood clusters experienced nontrivial declines in normal activity levels during and after the event. Additionally, the negative resilience capacity values, measured by the area under the curve (AUC) for each cluster, represent decreasing activity levels, and thus quantify the relative disruption caused by the event in these neighborhoods.

**Table 1:** Measurement of mean impact, time to recovery, and resilience capacity for each cluster.

Neighborhood cluster	# Of neighborhoods	Impact	Time to recovery	Resilience capacity
Group 4—Shelter-in-place (red)	958	0.37	2 weeks	5.67
Group 3—Stable (gray)	592	0.10	5 days	0.09
Group 2—Distressed (sky blue)	212	0.34	2 weeks	−3.65
Group 1—Abandoned (dark blue)	133	0.48	>3 weeks	−9.86

We present neighborhood cluster characteristics in Table 2 and find clear disparities based on measured community resilience capacity. Many neighborhoods in Group 1, which we label “abandoned”, are located in flood prone areas (~45% of the land area is within a 500-year floodplain), indicating a higher exposure risk to catastrophic flooding and infrastructural vulnerability. In fact, the southwest part of the greater Houston area near the Addicks and Barker Reservoirs was home to some of the most severely impacted neighborhoods (e.g., the Energy Corridor), with many homes flooded and streets impassable for several weeks^51^. While Group 1 exhibits physical and topographical vulnerabilities to flooding, these neighborhoods are found to have the highest household median income, a result of the significant presence of oil and gas sector employees. In contrast, Group 2 represents socio-economically vulnerable communities characterized by lower household incomes, higher unemployment rates, fewer homeowners, and a larger share of minority population (see Methods for details). These neighborhoods, which we label “distressed”, experienced the second-largest impact, with activity levels falling 34% below pre-hurricane levels. Furthermore, post-hurricane activity levels do not fully return to pre-hurricane levels during the study period, indicating time-invariant impacts that fundamentally shift community activity^18,19^. It is possible that the effects of the hurricane in these communities, such as severe property damage, resultant financial burdens, deterioration of infrastructure, or health-related impacts, resulted in extended recovery periods beyond the study timeframe or permanent changes in neighborhood composition. Group 3 (“stable”) neighborhoods maintain relatively constant levels of community activity, meaning the number of residents staying in these neighborhoods is consistent before, during, and after the event. These communities have lower proportions of racial and ethnic minorities, higher median income, and significantly higher homeownership rates than Group 2 neighborhoods, although their flood risk is similar (Table 2). The observed difference in mobility behavior supports previous research on the factors that influence hurricane evacuation decisions, with homeowners being less likely to evacuate in order to protect their property^36–39^. Activity patterns for Group 4 (“shelter-in-place”) neighborhoods form a positive bell-shaped curve, representing an increase in local activity above pre-event equilibrium. This cluster is composed of the least vulnerable neighborhoods with the second highest household incomes and largest share of homeowners and single-family homes (Table 2), while having the lowest relative flood risk. Some of those neighborhoods with increased residential activity levels as compared to the pre-hurricane period are also areas where large emergency shelters are located. The measured magnitude of impact of the hurricane for this group is 0.37 and the resilience capacity is positive (5.67), indicating that these neighborhoods experienced increases in residential activity and had the capacity to accommodate people for evacuation purposes from other communities.

**Table 2:** Neighborhood cluster characteristics.

Feature	4—Shelter-in-place	3—Stable	2—Distressed	1—Abandoned
	*n* = 958	*n* = 592	*n* = 212	*n* = 133
Demographic and socioeconomic features				
Black (%)	0.16 (0.18)	0.17 (0.18)	**0.20 (0.23)**	0.12 (0.14)
Hispanic (%)	0.38 (0.24)	0.39 (0.23)	**0.44 (0.26)**	0.33 (0.23)
Limited English speakers (%)	0.10 (0.11)	0.11 (0.11)	**0.15 (0.15)**	0.09 (0.12)
Educational attainment (College degree)	0.10 (0.09)	0.11 (0.10)	0.10 (0.11)	**0.15 (0.13)**
Educational attainment (High school degree)	0.23 (0.08)	0.23 (0.09)	**0.25 (0.10)**	0.19 (0.10)
Median income (USD)	$75,543 ($37,244)	$71,718 ($36,951)	$61,157 ($35,631)	**$82,126 ($42,390)**
Unemployment rate	0.06 (0.03)	0.06 (0.04)	**0.07 (0.04)**	0.05 (0.03)
Households without health insurance (%)	0.18 (0.11)	0.19 (0.11)	**0.23 (0.12)**	0.16 (0.11)
Households with food stamps (%)	0.11 (0.09)	0.12 (0.09)	**0.14 (0.10)**	0.09 (0.09)
Households without internet (%)	0.15 (0.12)	0.17 (0.13)	**0.22 (0.15)**	0.14 (0.13)
Homeowners (%)	**0.68 (0.20)**	0.62 (0.22)	0.54 (0.24)	0.63 (0.23)
Households living in mobile homes (%)	0.03 (0.07)	0.03 (0.06)	**0.05 (0.10)**	0.03 (0.07)
Land use and topographical features				
Median building age (years)	32 (16)	35 (56)	**39 (14)**	35 (14)
Median number of rooms	**6.01 (1.16)**	5.71 (1.22)	5.24 (1.11)	5.84 (1.27)
Vacancy rate (%)	0.07 (0.05)	0.08 (0.05)	**0.10 (0.05)**	0.09 (0.06)
Multifamily housing (%)	0.02 (0.03)	0.03 (0.05)	**0.04 (0.08)**	0.03 (0.05)
Lower elevation (% of land area)	0.07 (0.16)	0.08 (0.17)	0.09 (0.17)	**0.13 (0.20)**
Floodplain (% of land area)	0.30 (0.26)	0.30 (0.28)	0.34 (0.32)	**0.45 (0.31)**

Statistically significant differences between groups are tested using one-way ANOVA (analysis of variance) and Tukey’s multi-comparison method. Mean values with standard deviation in parentheses; highest values across the groups in bold.

**Fig. 2:**
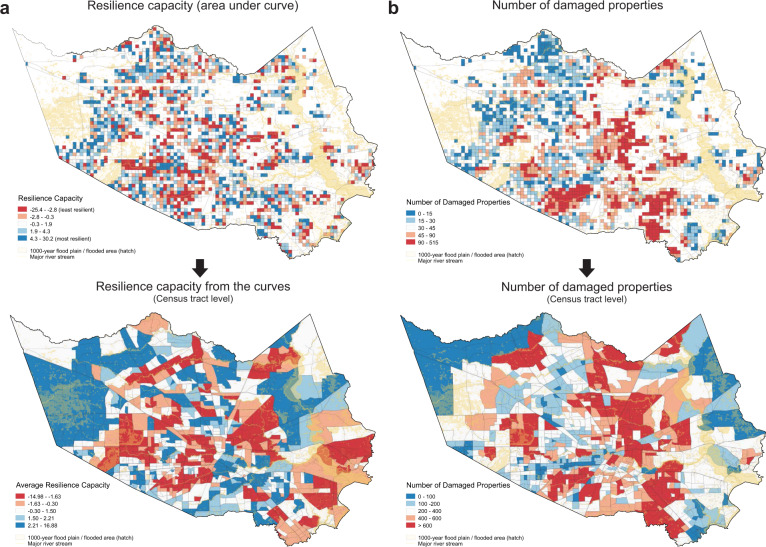
Validation of neighborhood resilience measure. **a** Neighborhood resilience capacity calculated as the area under the curve (AUC) represented in (**a**). Top is grid cell and bottom is aggregation to the census tract. Red colored neighborhoods are less resilient, while blue colored cells are more resilient. **b** The number of damaged properties based on the Federal Emergency Management Agency (FEMA) disaster assistance application data are illustrated at two different spatial resolutions (grid cell and census tract). Residents in red colored areas experienced more severe property damage.

As a validation of our resilience capacity measure, we use Federal Emergency Management Agency (FEMA) disaster assistance application data to correlate property damage with community resilience capacity. As shown in Fig. 2b, the number of verified damaged properties during Harvey in Group 1 neighborhoods, which include the most physically vulnerable communities to flooding, is 151 per 1000 households, which is 44% more than the least impacted group. This may help to explain why post-event neighborhood activity levels remain 20% below pre-event levels (Fig. 1a), indicating that evacuees from these communities may not have been able to return home within the study period due to widespread property damage. The number of damaged properties is plotted against the community resilience capacity computed by the AUC in Supplementary Fig. 4, with linear best fit line (*p* value = −0.37). The negative correlation indicates that neighborhoods with higher resilience capacity experienced fewer damaged properties. We caveat this finding with the acknowledgement of self-selection bias caused by individuals choosing to apply for federal assistance. Therefore, damage claims may skew toward homeowners and lower-income households that would have greater financial need.

### The evacuation divide: mobility and travel pattern of evacuees

To identify evacuation patterns for individual neighborhoods, we select 51,020 active users present throughout the study period who (1) maintain a residential location in Houston and (2) stay in their home neighborhood (grid cell) before Harvey was forecast. As the Houston city government had not yet officially declared an evacuation order, evacuees made decisions based on their own risk tolerance and financial and housing context. Therefore, the analysis accounts for those preemptively evacuating and those later forced to evacuate due to damage from the storm. We conduct a network analysis based on grid cell origin-destination pairs during the study period at three spatial scales: within Harris County, out of Harris County but within the state of Texas, and across the United States (see Materials and Methods for details).

Approximately 82.9% of users (42,277) stayed in their home neighborhood during Harvey and in the month afterwards (after August 31), while ~11% of the analyzed residents (5322) left their home neighborhoods during the impact period (the remainder were inactive after the event). Of those who evacuated, 68.1% (3624) stayed within the Houston Metropolitan area, 14.2% (758) traveled to another location within Texas, and 17.7% (940) left to other states across the country, with a majority traveling to Louisiana, New Mexico, and California. This finding supports results found in previous literature using post-disaster surveys and interviews^41^.

**Fig. 3:**
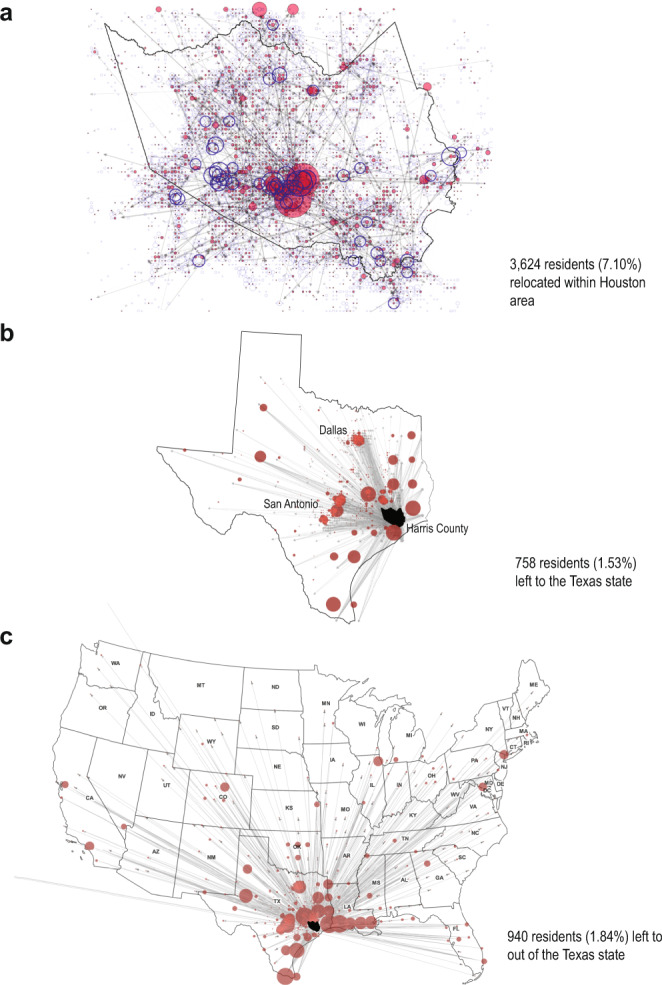
Disparities in evacuation patterns during Hurricane Harvey. **a** 3624 residents (7.10% of the total sample) left their home neighborhood, but stayed within the Houston area. Blue dots denote the outbound number of people and red dots denote the inbound number of people for a given neighborhood. Many people in the west area near reservoirs were forced to leave their home due to flooding during and after Harvey. People who left their neighborhood mostly evacuated to the Downtown area where multiple mega-shelters were located (**b**) 758 people (1.53% of the total sample) traveled to other parts of Texas (outside of the greater Houston area). Major destinations of those evacuees are College station, San Antonio, and Dallas where additional shelter programs were operating. **c** 940 people (1.84% of the total sample) left the Houston area to other states including Louisiana, New Mexico, and California. Most evacuees traveling out of Texas headed to Louisiana, the nearest neighboring state.

Focusing on the differences among the three different evacuation destination geographies—within Houston, within Texas, and out of Texas—we visually represent a network graph of evacuation patterns and associated household characteristics of evacuee neighborhoods in Fig. 3 and Supplementary Fig. 5. We observe distinct spatial patterns of origins and destinations for each evacuee group associated with evacuees’ socioeconomic status (Table 3). First, evacuees staying within the Houston area are more likely to be from the Distressed and Abandoned neighborhood groups, particularly in communities around Downtown Houston and the western edge of Downtown close to the reservoirs where most neighborhoods were impacted by flooding. The mean travel distance is found to be twelve (12) miles and the major destinations are located in the Downtown area proximate to evacuation shelters. Consistent with the characteristics of the neighborhoods in Groups 1 and 2, these evacuees are shown to be predominantly lower-income, minority and vulnerable households, with significantly higher proportions of residents without health insurance. On the other hand, evacuees who left Houston and relocated either within Texas or to other states traveled, on average, 140 miles and 540 miles, respectively, and had the means to wait out the storm in areas unaffected by it. These evacuee groups are composed of relatively higher income, homeowner, non-Hispanic white households with vehicles, and were more likely to be from neighborhoods in the Stable or Shelter-in-place groups. Additionally, although there was no official evacuation order, residents living along the eastern edge of Harris County are found to proactively leave their communities and travel to Louisiana because their neighborhoods are located within evacuation zones, reinforcing findings from previous literature about evacuation decision-making based on behavioral awareness during a hurricane event^52^.

**Table 3:** Disparities in disaster response and evacuation patterns across the four cluster groups.

Origin group	Sample size	Disaster response			Where evacuees go		
		Stay home	Evacuate	Inactive (No ping)	Harris County	Texas (outside of Harris County)	US (outside of Texas)
Shelter-in-place	9953	8493 (85.33%)	837 (8.41%)	623 (6.26%)	552 (65.95%)	133 (15.89%)	152 (18.16%)
Stable	16,506	13,936 (84.43%)	1506 (9.12%)	1064 (6.45%)	1008 (66.93%)	220 (14.61%)	278 (18.46%)
Distressed	4187	3338 (79.72%)	559 (13.35%)	290 (6.93%)	381 (68.16%)	57 (10.20%)	121 (21.65%)
Abandoned	1960	1320 (67.35%)	438 (22.35%)	2020 (10.31%)	343 (78.31%)	49 (11.19%)	46 (10.50%)

This table summarizes evacuation destinations based on residents’ origin (home neighborhood) associated with neighborhood activity clusters.

## Discussion

We present a methodology to quantify neighborhood-level evacuation and recovery patterns in Houston during Hurricane Harvey by analyzing large-scale mobility data. Mobility datasets have significant advantages for disaster management and planning, particularly as a complement to traditional data sources, such as post-event surveys and qualitative methods. While conventional data and methods can provide a rich resource for contextual information from small samples and limited geographical areas, the low cost and high coverage of geolocation data offer new opportunities to identify, model, and, ultimately, understand urban dynamics at scale. An important component of our approach is its generalizability and scalability: using the same or similar dataset, the methodology can be applied to other extreme or unexpected events, beyond natural disasters. For decision makers, these data can be used to better understand patterns and anomalies in human behavior in response to any number of shocks and to evaluate the impact of policy interventions in near-real-time.

Of particular concern, we find clear disparities in disaster response behaviors and resilience capacities across neighborhoods associated with their demographic, socioeconomic, and topographical characteristics. Mobility patterns during the hurricane are clustered into distinct neighborhood groups, demonstrating that predominantly low-income and minority neighborhoods are most impacted by the hurricane while least able to evacuate to safer areas outside of the impact area. When comparing neighborhoods with similar flood risk, communities with higher proportions of racial and ethnic minorities and renter households experienced a 37% decline in local activity levels, a shock that persisted into the post-event period. These communities are least able to withstand the financial and health impacts of the disaster, highlighted by the troubling fact that approximately one-quarter of residents in the Distressed neighborhood group lack health insurance. While it is often assumed that income and racial disparities result in worse outcomes for poor, minority communities, our method creates new opportunities to understand the dynamics of these impacts and take action to support vulnerable areas. Our work can help urban decision makers develop and implement data-driven resilience and emergency planning strategies that account for localized variations in risk and resilience capacity. Using large-scale mobility data enables proactive monitoring of community activity before and during a disaster such that the impact to neighborhoods can be evaluated in near-real-time. Our research can be expanded to post-event studies connected with long-term neighborhood recovery patterns and permanent migration patterns caused by natural disasters and global climate change. In addition to the ex post analysis of disasters and other anomalous events, rapid impact assessment based on observed resilience capacities provides another tool for local governments to prioritize the equitable allocation of resources, such as optimizing shelter locations and evacuation routing, targeting outreach to at-risk populations, and aiding more vulnerable neighborhoods.

## Methods

### Smartphone geolocation data and preprocessing

We use anonymized mobile device geolocation data from VenPath, Inc.—a data marketplace company providing mobile application data and business analytics services extracted from more than 200 various mobile applications and covering more than 120 million devices every month across the U.S. The initial dataset is 23TB of compressed comma-separated-value files (CSV) covering the period from June 2016 through October 2017 and contains more than 320 billion data points. Each data point represents a ping from a mobile device identified by an anonymized advertisement ID (*ad_id*), application ID, IP address of the network (if applicable), timestamp in unix format, device make and model, operating system and version, device location in (geographic coordinates—latitude and longitude), and the accuracy of the geolocation. Using *ad_id* as a key, one is able to link application activity and movement of the device in both time and space.

The sheer size of the data and confidentiality concerns pose nontrivial challenges in data management from both computation and privacy perspectives. Data were managed in accordance with NYU Institutional Review Board approval IRB-FY2018-1645 and stored and accessed in a secured environment at New York University’s Center for Urban Science and Progress (NYU CUSP) Research Computing Facility (RCF), which is equipped with High Performance Computing (HPC) infrastructure, controlled access, and restricted connectivity. Data processing are conducted using Python version 3.7 and Apache PySpark version 2.4. Each observation was pre-processed to standardize the format between various reporting applications. The *ad_id* encoding was standardized to all uppercase characters and devices with a generic *ad_id* (e.g., “00000000-0000-0000-0000-000000000000” for randomized Apple devices) were excluded. In order to standardize reporting frequencies across various applications and further obscure users’ exact location, ping coordinates were averaged over a 5 min time interval and aggregated to grid cell, census tract, and zipcode geographies. For this analysis, we extract a subset of the data for the period directly preceding and following Hurricane Harvey spanning 2 months (2017-08-01 to 2017-09-30) and falling within the bounding box defined by −94.8285W and −95.9065W of longitude and 29.4492N and 30.0685N of latitude encompassing the Greater Houston Area. We also localize timestamps to the Central Daylight Time (GMT-5) zone. Since the analysis focuses on changes in daily residency locations, we also filter out pings originating from highways or major roads to eliminate vehicular activity within a given census tract or grid cell, as that activity could skew measures of residential activity within a given neighborhood. The resultant dataset consists of a total of 829,350 unique devices.

Ancillary data listed in Supplementary Table 1 are used for contextual analysis and validation of the results. Data from the 2017 5-year estimate U.S. Census Bureau American Community Survey (ACS) are used to obtain census tract geometries and relevant socioeconomic, housing, and demographic characteristics. The locations of major roads are identified to flag observations associated solely with vehicular travel. Other relevant datasets include United States Geological Survey (USGS) elevation data along with FEMA floodplain areas, shelter locations, and Evacuation Zone spatial boundaries.

### Quantifying community-level spatiotemporal evacuation and recovery patterns

We develop a four-step methodology to quantify impact, evacuation, and recovery patterns across neighborhoods before, during, and after Hurricane Harvey. The geographical scope of this analysis is Harris county (1777*mi*^2^), covering the central section of the Houston Metropolitan Area. This research uses the Python programming language (version 3.7) and Quantum GIS version 3.4 Madeira to implement geospatial analyses and machine learning applications.

#### Step 1: Identifying users’ daily residence activity areas

Daily residence activity areas for each of the 829,350 users during the study period are identified based on an *argmax* value of pings for each individual user. Each ping is represented by the vector < *p*, *t*_*p*_ > , where *p* denotes a device’s location at time *t*_*p*_. We use an ~1 km by 1 km grid cell (0.6214 mile × 0.6214 mile) as the areal unit of *p* to preserve privacy and minimize re-identification risks. The residence activity area of each user is specified as:

1 $${H}_{u,{d}_{n}}\,=\,argmax({\forall }_{p}\,\in\, {P}_{u}| {t}_{p}\,\in\, {R}_{u}:\sum {R}_{u}(i,p))$$

where ${H}_{(u,{d}_{n})}$ is the main activity area of user *u* on day *n*, and *P*_*u*_ is the set of all grid cell locations with pings by user *u*. In other words, a daily residence activity location (home/shelter/hotel/temporary residence, etc.) for each user is defined by the grid cell with the most frequent pings for that user for a given day. For weekdays, we only use pings from 8 p.m. to 7 a.m., when people are more likely to be present at their location of residence. The results provide a daily grid cell home location for each device that can be used to analyze changes in residence patterns over the study period.

#### Step 2: Quantifying the change in the number of users staying in a neighborhood

Based on the output from step 1, we calculate a baseline for neighborhood activity levels as the average number of users in a given grid cell before August 16th. We assume that activity in this period is not affected by Hurricane Harvey so that the residential population of a given neighborhood is relatively stable. For the remaining period, we calculate a percentile distance from the average value to the number of daily users in a given grid cell, specified as:

2 $${D}_{N{d}_{n}}\,=\,\frac{{U}_{N{d}_{n}}\,-\,{U}_{NA}}{{U}_{NA}}$$

where ${D}_{N{d}_{n}}$ is the percentile distance between the average number of users to the number of users on a given day *n*, ${U}_{N{d}_{n}}$ is the number of users in grid cell *N* on a day *n*, and *U*_*NA*_ is the average number of users in grid cell *N* during the pre-hurricane equilibrium (2017-08-01 to 2017-08-16). These time series values represent the changing pattern of the number of users staying in a given neighborhood (grid cell), providing a measure of the variance in residential activity levels over the time period.

#### Step 3: Classifying neighborhoods based on disaster response and recovery patterns

In order to identify disaster response patterns, we first decompose observed values from step 2 based on a 3-day moving average to extract a trend from which we implement an agglomerative clustering algorithm. An agglomerative clustering algorithm is a widely-used bottom-up hierarchical clustering method based on mathematical distance. The algorithm starts by considering each data point as a single cluster and, at each iteration, similar clusters are merged with neighboring clusters based on the proximity matrix until all clusters are merged into one cluster^53^. An input variable is a vector of the trend of the number of users in each neighborhood from August 4 through September 26 (the shorter time range results from the moving average transformation). The optimized number of clusters is selected by the hierarchical clustering dendrogram based on similarities/dissimilarities of observations, aiming to minimize the variance within the clusters, while maximizing the variance between the different groups. We use Ward’s metric to calculate distance between clusters *C*_*i*_ and *C*_*j*_, specified as:

3 $${D}_{w}({C}_{i},{C}_{j})\,=\,\mathop{\sum} _{x\,\in\, {C}_{i}}{(x\,-\,{r}_{i})}^{2}\,+\,\mathop{\sum} _{x\,\in\, {C}_{j}}{(x\,-\,{r}_{j})}^{2}\,-\,\mathop{\sum} _{x\,\in\, {C}_{ij}}{(x\,-\,{r}_{ij})}^{2}$$

where *C*_*ij*_ is a merging the two clusters *C*_*i*_ and *C*_*j*_, *r*_*i*_, *r*_*j*_, and *r*_*ij*_ are the centroids of *C*_*i*_, *C*_*j*_, and *C*_*ij*_ respectively.

#### Step 4: Measurement of community resilience capacity

Once we have identified neighborhood-level hurricane response activity patterns, the magnitude of impact and time-to-recovery can be measured as described in Supplementary Fig. 2. The magnitude of impact is measured as the maximum depth or maximum height of the community activity curve during the hurricane impact period. Time-to-recovery is measured as the number of days between the date of peak amplitude and the date when the neighborhood reaches post-event activity equilibrium. To this end, we introduce a unified measure of resilience capacity that reflects both a magnitude of impact and recovery duration, calculated as the area under/over the curve (shadowed in Supplementary Fig. 2) using the formula:

4 $$R{C}_{N}\,=\,\mathop{\int}\nolimits_{\!\!\!\!\!{t}_{0}}^{{t}_{2}}{A}_{equilibrium}\,-\,{A}_{N}(t)dt$$

where *t*_0_ is the date of maximum impact, *t*_2_ is time to reach post-event equilibrium, and *A*_*N*_(*t*) is a function of community activity for neighborhood *N* over time. Since resilience capacity must account for both the magnitude of a shock and the time to return to pre-event equilibrium activity levels, our approach can provide a generalizable measure of community resilience that extends beyond static indices of impact or recovery. After clustering, we integrate and spatially join other data sources to estimate correlations between community resilience capacity and neighborhood characteristics described in Supplementary Table 1. All of the ancillary data are publicly available and extracted from open data platforms. The neighborhood clustering outputs are contextualized to understand the profile of cluster groups based on neighborhood social, geophysical, and economic characteristics, such as evacuation zone and floodplain areas, topography, land use, and household demographic and socioeconomic attributes.

### Validation of community resilience capacity

FEMA disaster assistance application data include the number of grant applications from homeowners and renters and the number of verified damaged properties that are eligible to receive federal grants based on inspections. We use data on the number of damaged properties, initially provided at zipcode aggregation, to estimate the actual neighborhood-level impacts from Hurricane Harvey. We calculate the number of damaged properties per household and multiply it by the number of households in a given grid cell to estimate the hurricane-related damage for each grid cell to match the spatial resolution of the activity measures.

### Mobility patterns of evacuees during and after Hurricane Harvey

In order to gain a better understanding of evacuees’ mobility patterns and disparities in evacuation behavior, we subset devices active in the Houston area during the direct impact period (between August 21, 2017 and August 31, 2017) and extract the top 25% most active users living in the Houston area and for whom geolocation data are available throughout the study period (51,020 unique devices). We then expand the geographical scope of our analysis to include all pings generated from those devices covering the entire territory of the United States in order to identify the full extent of evacuation patterns.

To define mobility behavior before, during, and after the hurricane, one needs to identify areas of activity for each device, namely home, work, and other places, such as recreation, entertainment, or shopping, as well as potential temporary locations of residence if displaced during the hurricane. Since behavior patterns vary significantly across devices, there is no predefined number of areas of activity. To define these areas, we use a density-based spatial clustering of applications with noise (DBSCAN) clustering algorithm^54^. The advantages of DBSCAN in this instance are (1) that it does not require a priori information about the number of clusters, (2) the clusters can be of arbitrary shape defined by the chosen distance measure, and (3) the algorithm is robust to outliers identified as noise (not belonging to any of the clusters). Two parameters required to be specified are the distance between the observation points (*ε*) and minimum number of points required to form a cluster (*n*). The algorithm defines a cluster *C* as a set of *density* − *connected* points within the *Eps* − *neighborhood* of a point *p*, denoted as *N*_*ε*_(*p*), where:

5 $${N}_{\varepsilon }(p)\,=\,\{q\,\in\, C| dist(p,q)\,\leqslant\, \varepsilon \}\,\wedge\, | C| \,\geqslant\, n$$

To identify the most common areas of activity for each *ad_id*, we perform DBSCAN in three dimensions: latitude, longitude, and time. Since the geographical extent for each device’s activity is unique and varies significantly, both latitude and longitude were rescaled to kilometers based on the size of the bounding box encompassing the full range of each user’s activity. Temporal clustering is based on hour of activity and was rescaled from a 0–288 range (the number of 5 min periods within 24 h) to 0–1. Using the rescaled values, we set the Euclidean distance parameter *ε* to 0.25, corresponding to 250 meters and 6 h for the spatial and temporal dimensions, respectively. The minimum point number (*n*) is dependent on the number of days the device was active. The resultant database contains the clusters of activity identified for each device, characterized by their size, average geographical location, list of dates when the cluster was activated, and the hourly distribution of activity within the cluster.

Using the hourly distribution of activity and the size of each cluster, we then identify the most probable home location for each user. In order to do so we apply a *k*-means clustering method of normalized hourly activity cluster profiles^53^. The algorithm classifies the set *n* of samples *x* into *k* nonoverlapping clusters *C*, of equal variance and described by the mean *μ*_*j*_, minimizing within-cluster sum-of-squares (*SS*) defined as:

6 $$SS\,=\,\mathop{\sum }\limits_{i\,=\,0}^{n}mi{n}_{{\mu }_{j}\,\in\, C}(\parallel {x}_{i}\,-\,{\mu }_{j}{\parallel }^{2})$$

Prior to clustering, the time series data are smoothed using an exponentially weighted moving average to remove noise from the ping observations. The results yield four common profiles that can be associated with different types of activity, particularly residential (with activity peaks in the early morning and later in the evening or throughout the day on weekends) and work locations (with most of activity occurring during traditional working hours and often extended into the evening). Each user’s pre-hurricane home location was assigned to the most commonly visited cluster of activity before Harvey from within the first two categories described above. We validate the results of home location against census tract level population from the U.S. Census Bureau ACS and obtain a 0.77 correlation coefficient. To track changes in daily home locations and potential evacuation behavior, a similar process was performed using only the clusters activated on that given day without constraint to cluster category, but with priority given to residential profiles.

We are concerned with socioeconomic and demographic disparities in evacuation behavior before, during, and after Hurricane Harvey. Based on the computed daily residence neighborhoods for active users, we create origin-destination pairs for each individual aggregated to the grid cell level based on the (1) initial (pre-event) home location and (2) the residential location during and after Harvey. We focus on the time period between August 23rd and September 2nd to identify evacuation locations for those that leave their home neighborhood. The origin-destination pairs generate an origin-destination matrix denoting a origin location, a destination location, and the number of users associated with a given origin-destination pair. In addition to the destination location (a centroid of a grid cell), we encode destination locations as one of four different types: home, within Houston, within Texas but outside of Houston, and outside of Texas. Evacuees are classified by their destination location and we compare their travel distances and destination locations as a function of origin neighborhood location demographic and socioeconomic characteristics (Supplementary Table 1).

### Reporting summary

Further information on research design is available in the Nature Research Reporting Summary linked to this article.

## Supplementary information

Supplementary Information

Reporting Summary

## Data Availability

The primary mobile phone geolocation data that support the findings of this study are available from VenPath, Inc., but restrictions apply to the availability of these data, which were used under data sharing agreement and are not publicly available. The aggregated data used for this analysis may be available from the authors upon reasonable request and with permission of VenPath, Inc. Figure 1 and Supplementary Fig. 2 are associated with the geolocation data source. Additional data needed to evaluate the analyses in the paper are described in Supplementary Table 1. All data related to this study may be requested from the corresponding author upon reasonable request and with permission of the data provider if data are not publicly available. Original Houston Harris County parcel level land use information data is available through the Texas Natural Resources Information System website (https://data.tnris.org/collection/2679b514-bb7b-409f-97f3-ee3879f34448). Data on Houston park and open space is available from the City of Houston GIS Open Data Portal (COHGIS) (https://cohgis-mycity.opendata.arcgis.com/datasets/coh-park-boundary), and Harris County evacuation zones from the Harris County Emergency Management (https://prepare.readyharris.org/evacuation-map). The U.S. topology information was obtained from the USGS National Elevation Dataset (NED 1/3 arc-second) (https://catalog.data.gov/dataset/national-elevation-dataset-ned-1-3-arc-second-downloadable-data-collection-national-geospatial), the national flood hazard layer and the disaster assistance appliation information from FEMA (https://www.fema.gov/flood-maps/products-tools/national-flood-hazard-layer and https://www.fema.gov/about/openfema/data-sets respectively), and the FEMA shelter locations through the Rice University Houston Urban Data Platform (https://www.kinderudp.org/#/datasetCatalog/va7b869ng5dv). Data on Texas major roads including highways is accessible through the Texas Department of Transportation and the Houston Department of Transportation (https://gis-txdot.opendata.arcgis.com/datasets/d4f7206d27af4358acb70cb1cc819d10_0 and https://cohgis-mycity.opendata.arcgis.com/datasets/coh-major-road respectively). All demographic and household socioeconomic data were retrieved from the U.S. ACS administrated by Census Bureau (https://www.census.gov/programs-surveys/acs/technical-documentation/table-and-geography-changes/2017/5-year.html).

## Code availability

All of the code used to process data and perform the analysis for this study, as well as the resulting models and figures are available in the dedicated GitHub repository—https://github.com/UrbanIntelligenceLab/measuring-inequality-in-community-resilience-to-natural-disasters under MIT License.
