# Supplementary material for: Measuring inequality in community resilience to natural disasters using large-scale mobility data
Source: Nat Commun. 2021 Mar 25;12:1870. doi: 10.1038/s41467-021-22160-w (PMC7994553; doi:10.1038/s41467-021-22160-w)
Supplement: Supplementary file 1 — Supplementary Information [file 41467_2021_22160_MOESM1_ESM.pdf]

Supplementary Information for

Measuring Inequality in Community Resilience to Natural Disasters Using Large-scale Mobility Data

Boyeong Hong[1], Bartosz J. Bonczak[1], Arpit Gupta[2], and Constantine E. Kontokosta[1,3,*]

[1]New York University, Marron Institute of Urban Management, 60 5th Avenue, New York, NY 10011

[2]New York University, Stern School of Business, 44 West 4th Street, New York, NY 10012

[3]New York University, Center for Urban Science and Progress, 370 Jay Street, Brooklyn, NY 11201

[*]Correspondence to: ckontokosta@nyu.edu

Content

- Supplementary Table 1: Additional data sources and descriptions.
- Supplementary Figure 1: Spatial distributions of ping locations in Houston.
- Supplementary Figure 2: Conceptual resilience curve and a measurement of resilience.
- Supplementary Figure 3: Timeseries of the percentile distance between the average number of users in their home neighborhood.
- Supplementary Figure 4: Scatter plot of average resilience capacity measured by area under/over curves versus the number of damaged properties per household.
- Supplementary Figure 5: Disparities in evacuation patterns and household characteristics.

Supplementary Table 1. Additional data sources and descriptions

Data	Size	Description	Spatial granularity	Data coverage	Source
Parcel level land use	1.3 GB	Houston Harris County parcel level land use information such as property owner, land use type, and location attributes etc.	Parcel	2018	Texas Natural Resources Information System
Park and open space	8 MB	Texas State parcel-based areas of parks and open spaces	Dissolved by park	2018	City of Houston GIS(COHGIS) Open Data Portal
Physical topology	1.1 GB	Physical topology associated information including elevation	Related polygon	2013	USGS National Elevation Dataset (NED - 1/3 arc-second)
National Flood Hazard Layer	11 GB	Geographical information of an area of land adjacent to watershed and valley with high likelihood of flooding	Related polygon	2018	Federal Emergency Management Agency
Evacuation zone	-	List of zipcodes of planned evacuation zones	-	2018	Harris County Emergency Management
FEMA shelters	28 MB	FEMA National Shelter System information with geotagged locations	GPS (X,Y)	2017	Rice University Houston Urban Data Platform
Major roads	650 MB	Texas statewide and Houston citywide major roadway information associated with spatial segments, including road type, number of lanes etc.	Road segment	2019	Texas Department of Transportation<br><br>Houston Department of Transportation
FEMA disaster assistance application	-	Federal aid for residents who suffered damage and losses from Hurricane Harvey	Zipcode	2017	Federal Emergency Management Agency
U.S. American Community Survey	20 MB	2017 5-year estimate. U.S. demographic and household socioeconomic characteristics data, such as population, age, race, income level, household type etc.	Census Tract	2017	Census Bureau (U.S. Department of Commerce)

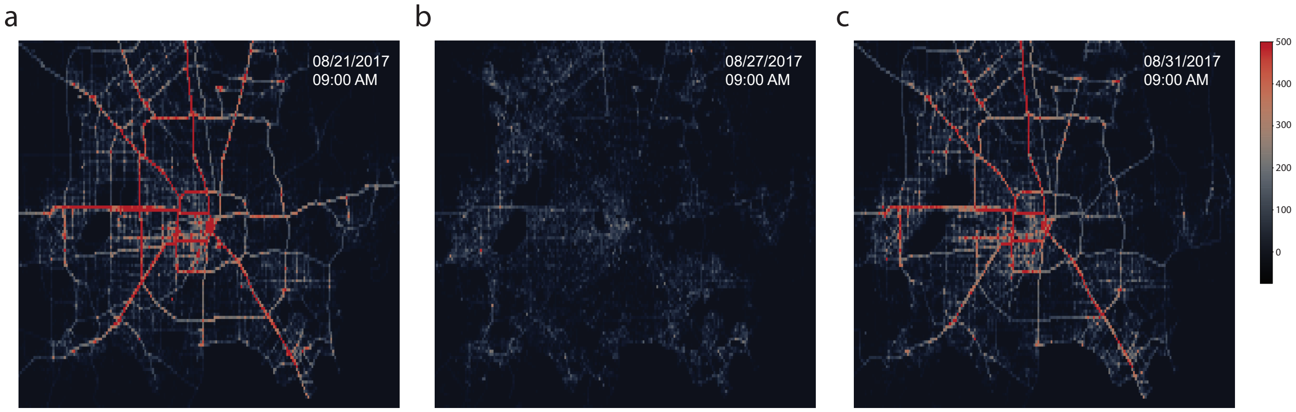

Supplementary Figure 1. Spatial distributions of ping locations in Houston. (a) Number of unique devices (prior to filtering vehicular traffic) by 1km $\times$ 1km grid cell on August 21st, 2017 at 9:00 AM before Harvey reaches the city. The Texas state government declared a state of emergency on August 23rd. (b) Hurricane Harvey hit the greater Houston area on August 25th and a significant decrease in activity levels can be seen on August 27th at 9:00 AM. (c) On August 31st, many neighborhood activities begin to return to pre-hurricane levels, but the intensity is still relatively less than pre-event levels (a).

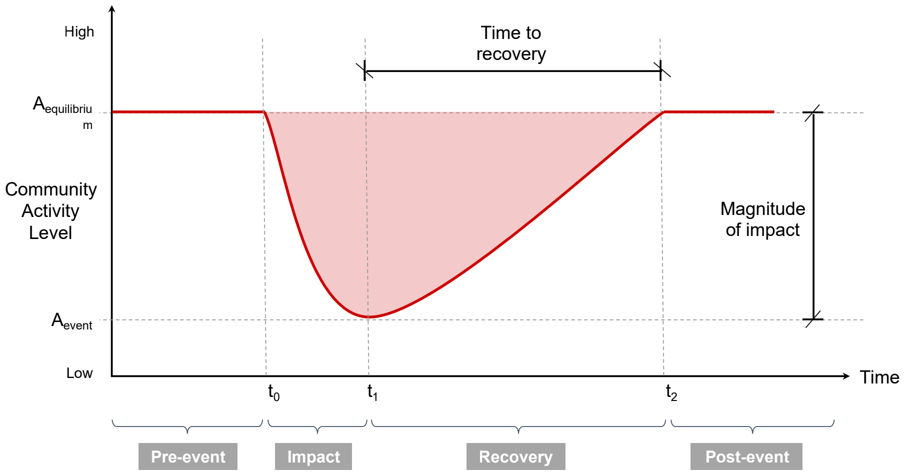

Supplementary Figure 2. Conceptual resilience curve and the measurement of resilience, adopted from Kontokosta and Malik (2018) and the National Association of Development Organization Research Foundation (2015).
The conceptual resilience curve consists of four phases: 1) pre-event, 2) impact, 3) recovery, and 4) post-event. The magnitude of impact is measured as the maximum depth or maximum height of the community activity curve, which is given by $A_{equilibrium}$ - A_{event}. The time-to-recovery represents the number of days between the maximum impact point and the post-event equilibrium, t_2-t_1. We introduce a measure of resilience capacity that reflects both the magnitude of impact and recovery duration, calculated as the shaded area under/over the curve.

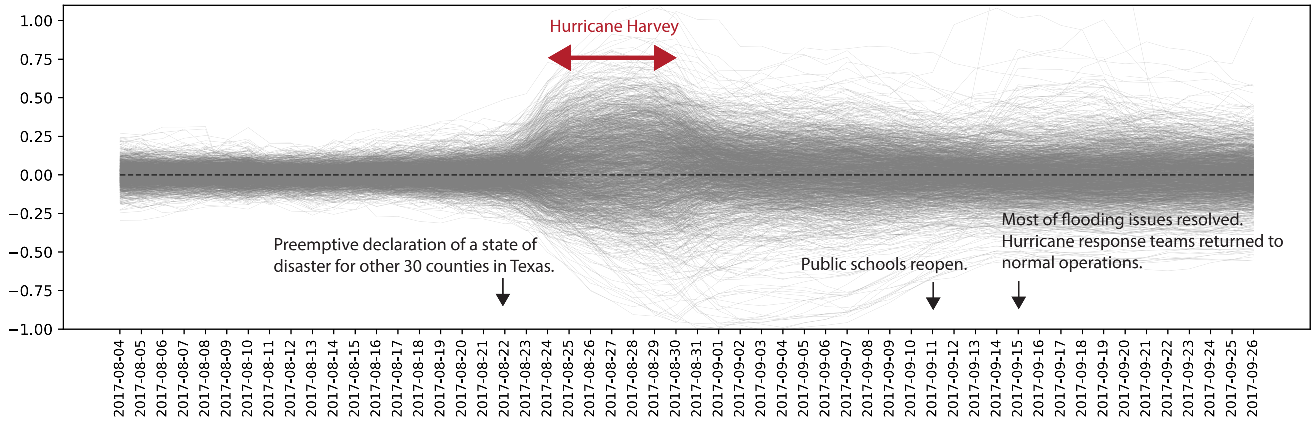

Supplementary Figure 3. Time series of the percentile distance between the mean number of users in their home neighborhood during the pre-hurricane period (August 1 to August 16) and the number of daily users staying in a given neighborhood over the event duration, using a three-day moving average. Hurricane Harvey hit the greater Houston area on August 25, 2017 and it lasted until August 30, 2017.

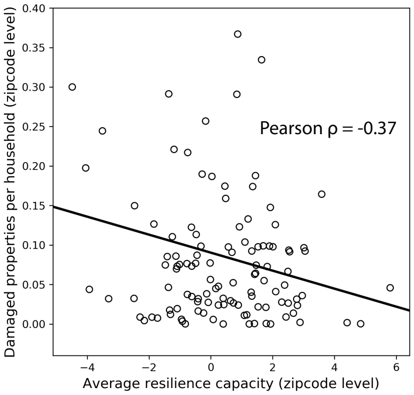

Supplementary Figure 4. Scatter plot of mean resilience capacity measured by the area under/over community resilience curves versus the number of damaged properties per household in that neighborhood. There is a statistically significant negative correlation between neighborhood resilience and this proxy for hurricane damage (ρ = -0.37). As Federal Emergency Management Agency (FEMA) data are provided at the zipcode level, the neighborhood resilience capacity is aggregated to zipcodes for the comparison.

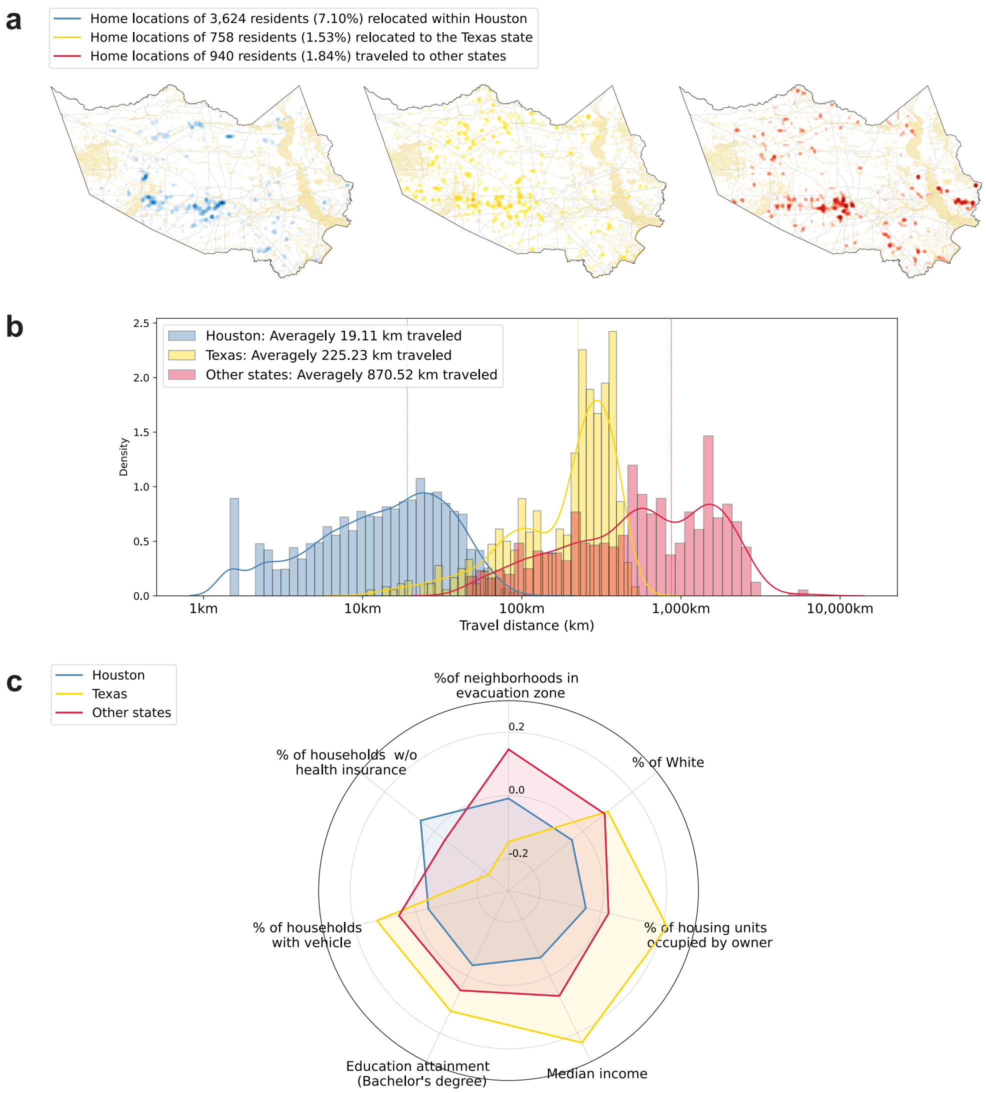

Supplementary Figure 5. Disparities in evacuation patterns and household characteristics
(a) From left to right, three groups denote evacuees relocating within Houston (blue), those traveling out of Houston but within the state of Texas (yellow), and those traveling to other states (red). (b) Distribution of travel distances for each evacuee group. The x-axis is log scale. (c) The radar plot illustrates the neighborhood demographic and socioeconomic characteristics of the three evacuee groups based on z-score normalized features. The citywide mean value is 0 and standard deviation is 1.
