# Supplementary material for: Measuring inequality in community resilience to natural disasters using large-scale mobility data
Source: Nat Commun. 2021 Mar 25;12:1870. doi: 10.1038/s41467-021-22160-w (PMC7994553; doi:10.1038/s41467-021-22160-w)
Supplement: Supplementary file 2 — Reporting Summary [file 41467_2021_22160_MOESM2_ESM.pdf]

nature research

Corresponding author(s): Constantine Kontokosta

Last updated by author(s): Jan 5, 2021

# Reporting Summary

Nature Research wishes to improve the reproducibility of the work that we publish. This form provides structure for consistency and transparency in reporting. For further information on Nature Research policies, see our Editorial Policies and the Editorial Policy Checklist.

## Statistics

For all statistical analyses, confirm that the following items are present in the figure legend, table legend, main text, or Methods section.

| n/a | Confirmed | |
|---|---|---|
| ☐ | ☒ | The exact sample size (*n*) for each experimental group/condition, given as a discrete number and unit of measurement |
| ☐ | ☒ | A statement on whether measurements were taken from distinct samples or whether the same sample was measured repeatedly |
| ☐ | ☒ | The statistical test(s) used AND whether they are one- or two-sided<br>*Only common tests should be described solely by name; describe more complex techniques in the Methods section.* |
| ☐ | ☒ | A description of all covariates tested |
| ☐ | ☒ | A description of any assumptions or corrections, such as tests of normality and adjustment for multiple comparisons |
| ☐ | ☒ | A full description of the statistical parameters including central tendency (e.g. means) or other basic estimates (e.g. regression coefficient) AND variation (e.g. standard deviation) or associated estimates of uncertainty (e.g. confidence intervals) |
| ☐ | ☒ | For null hypothesis testing, the test statistic (e.g. *F*, *t*, *r*) with confidence intervals, effect sizes, degrees of freedom and *P* value noted<br>*Give P values as exact values whenever suitable.* |
| ☒ | ☐ | For Bayesian analysis, information on the choice of priors and Markov chain Monte Carlo settings |
| ☐ | ☒ | For hierarchical and complex designs, identification of the appropriate level for tests and full reporting of outcomes |
| ☐ | ☒ | Estimates of effect sizes (e.g. Cohen's *d*, Pearson's *r*), indicating how they were calculated |

*Our web collection on statistics for biologists contains articles on many of the points above.*

## Software and code

Policy information about availability of computer code

| | |
|---|---|
| Data collection | The mobile phone geolocation data used for this study were collected by VenPath, Inc. - a data marketplace company, from more than 200 mobile applications covering more than 120 million devices every month across the U.S. The data were provided to the authors using AWS S3 cloud storage service and transferred to the New York University Center for Urban Science and Progress Research Computing Facility (RCF) using an AWS Snowball solution. RCF is equipped in High Performance Computing (HPC) infrastructure facilitating data processing efforts. |
| Data analysis | All analyses were conducted using Python version 3.7, Apache PySpark version 2.4, and QGIS version 3.4 Madeira. All of the code used to process data and perform the analysis for this study as well as the resulting models and figures are available in the dedicated GitHub repository - https://github.com/UrbanIntelligenceLab/large-scale-mobility-behavior-and-community-resilience under MIT License. |

For manuscripts utilizing custom algorithms or software that are central to the research but not yet described in published literature, software must be made available to editors and reviewers. We strongly encourage code deposition in a community repository (e.g. GitHub). See the Nature Research guidelines for submitting code & software for further information.

## Data

Policy information about availability of data

All manuscripts must include a data availability statement. This statement should provide the following information, where applicable:

- Accession codes, unique identifiers, or web links for publicly available datasets
- A list of figures that have associated raw data
- A description of any restrictions on data availability

The primary mobile phone geolocation data that support the findings of this study are available from VenPath, Inc., but restrictions apply to the availability of these data, which were used under data sharing agreement, and so are not publicly available. Data are however available from the authors upon reasonable request and with permission of VenPath, Inc.

Figure 1 and Figure S2 are associated with the raw data source. Additional data needed to evaluate the analyses in the paper are described in Supplementary Materials Table S1 and are publicly available at the web links shown below. All data related to the current study may be requested from the corresponding author upon reasonable request and with permission of the data provider if data are not publicly available.

Publicly available datasets used in the study:
- Houston Harris County parcel level land use information data obtained from Texas Natural Resources Information System website at https://data.tnris.org/collection/2679b514-bb7b-409f-97f3-ee3879f34448
- Houston pParks and Open Spaces obtained from the City of Houston GIS Open Data Portal (COHGIS) at https://cohgis-mycity.opendata.arcgis.com/datasets/coh-park-boundary
- Harris County Evacuation Zone obtained from the Harris County Emergency Management at https://prepare.readyharris.org/evacuation-map
- The U.S. Physical Topology Information obtained from the USGS National Elevation Dataset (NED 1/3 arc-second) at https://catalog.data.gov/dataset/national-elevation-dataset-ned-1-3-arc-second-downloadable-data-collection-national-geospatial
- The National Flood Hazard Layer and The Disaster Assistance Application Information obtained through the Federal Emergency Management Agency (FEMA)at https://www.fema.gov/flood-maps/products-tools/national-flood-hazard-layer and https://www.fema.gov/about/openfema/data-sets respectively
- FEMA Shelter Locations dataset was obtained through the Rice University's Houston Urban Data Platform at https://www.kinderudp.org/#/datasetCatalog/va7b869ng5dv
- Texas Major Roads was obtained through the Texas Department of Transportation and the Houston Department of Transportation at https://gis-txdot.opendata.arcgis.com/datasets/d4f7206d27af4358acb70cb1cc819d10_0 and https://cohgis-mycity.opendata.arcgis.com/datasets/coh-major-road respectively
- Demographic and household socioeconomic data were retrieved from the U.S. American Community Survey administrated by Census Bureau at https://www.census.gov/programs-surveys/acs/technical-documentation/table-and-geography-changes/2017/5-year.html

# Field-specific reporting

Please select the one below that is the best fit for your research. If you are not sure, read the appropriate sections before making your selection.

☐ Life sciences ☒ Behavioural & social sciences ☐ Ecological, evolutionary & environmental sciences

For a reference copy of the document with all sections, see nature.com/documents/nr-reporting-summary-flat.pdf

# Behavioural & social sciences study design

All studies must disclose on these points even when the disclosure is negative.

| | |
|---|---|
| Study description | The study involves quantitative research using mobile phone geolocation data and machine learning algorithms to evaluate the impact and evacuation patterns during the Hurricane Harvey that hit the Houston, TX area in August 2017. |
| Research sample | The study is based on anonymized mobile phone geolocation data provided by VenPath, Inc. extracted from more than 200 various mobile applications. Raw data includes more than 120 million unique devices every month across the United States. For the purpose of this study, the sample was subset to devices active within Houston, TX area in the period directly before and after Hurricane Harvey's impact, as described in the Methods section. After data processing, the sample data for the study includes 1,229,270 unique devices, representing approximately 43.2% of the total population of the Houston, TX area. |
| Sampling strategy | The study extracts the subset of the raw data spanning a two-month period from August 2017 through September 2017 and use all devices active at least once within the geographical bounding box of Houston, defined as 94.8285W and -95.9065W of longitude and 29.4492N and 30.0685N of latitude. |
| Data collection | VenPath, Inc. collects anonymized geolocation data from more than 200 various mobile applications across 120 million unique devices in the U.S. The original location data was passively collected as a result of mobile application activity by the user, saved in the applications' database and then provided to VenPath, Inc. The data made available for this study cover the period from June 2016 through October 2017, and were distributed to the researchers through AWS cloud services and New York University Center for Urban Science and Progress Research Computing Facility in accordance with NYU Institutional Review Board approval due to the size of the data and confidentiality concerns. Data collection was independent from the study and as such the researchers were not involved in the experimental conditions of its acquisition. |
| Timing | The study uses data collected between August 1st, 2017 and September 30th, 2017. |
| Data exclusions | Any observation with a generic ID (eg. "00000000-0000-0000-0000-000000000000" for randomized Apple devices) were excluded as randomization makes it impossible to link device movements over time since all of the randomized devices share the same generic identifier. The activity of excluded randomized devices accounts for 133,616,697 data points or 9.02% of the raw data. This exclusion was established after initial data investigation and based on existing literature on the subject matter. |
| Non-participation | Not applicable; this study is observational based on the data collected through various mobile applications. |
| Randomization | This study is observational and no randomization was used. |

# Reporting for specific materials, systems and methods

We require information from authors about some types of materials, experimental systems and methods used in many studies. Here, indicate whether each material, system or method listed is relevant to your study. If you are not sure if a list item applies to your research, read the appropriate section before selecting a response.

## Materials & experimental systems

| n/a | Involved in the study |
| --- | --- |
| ☒ | ☐ Antibodies |
| ☒ | ☐ Eukaryotic cell lines |
| ☒ | ☐ Palaeontology and archaeology |
| ☒ | ☐ Animals and other organisms |
| ☐ | ☒ Human research participants |
| ☒ | ☐ Clinical data |
| ☒ | ☐ Dual use research of concern |

## Methods

| n/a | Involved in the study |
| --- | --- |
| ☒ | ☐ ChIP-seq |
| ☒ | ☐ Flow cytometry |
| ☒ | ☐ MRI-based neuroimaging |

# Human research participants

Policy information about studies involving human research participants

| | |
| --- | --- |
| Population characteristics | The population selection for the purpose of this study was based on mobile application activity on applications aggregated by VenPath, Inc. during the study period within the study area described above. These are anonymized data and the gender and age composition of the participants (or any other demographic characteristics) is not known. |
| Recruitment | There was no recruitment procedure involved as the study was conducted based on already collected anonymized mobile phone geolocation data extracted from devices using mobile applications aggregated by VenPath, Inc. As the data are generated by mobile application activity, there is a risk of under-representation of some demographic groups, such as elderly populations, with a bias towards younger and digitally-connected populations. |
| Ethics oversight | The study procedure was designed in accordance with and approved by the NYU Institutional Review Board (approval IRB-FY2018-1645). |

Note that full information on the approval of the study protocol must also be provided in the manuscript.
